# ﻿Complete chloroplast genome sequence of *Rhododendronmariesii* and comparative genomics of related species in the family Ericaeae

**DOI:** 10.3897/compcytogen.17.101427

**Published:** 2023-08-18

**Authors:** Zhiliang Li, Zhiwei Huang, Xuchun Wan, Jiaojun Yu, Hongjin Dong, Jialiang Zhang, Chunyu Zhang, Shuzhen Wang

**Affiliations:** 1 College of Biology and Agricultural Resources, Huanggang Normal University, Huanggang, 438000, Hubei Province, China Huanggang Normal University Huanggang China; 2 College of Plant Science & Technology, Huazhong Agricultural University, Wuhan, 430070, Hubei Province, China Huazhong Agricultural University Wuhan China

**Keywords:** chloroplast genome, comparative genomics, conservation genetics, phylogeny, *Rhododendronmariesii*

## Abstract

*Rhododendronmariesii* Hemsley et Wilson, 1907, a typical member of the family Ericaeae, possesses valuable medicinal and horticultural properties. In this research, the complete chloroplast (cp) genome of *R.mariesii* was sequenced and assembled, which proved to be a typical quadripartite structure with the length of 203,480 bp. In particular, the lengths of the large single copy region (LSC), small single copy region (SSC), and inverted repeat regions (IR) were 113,715 bp, 7,953 bp, and 40,918 bp, respectively. Among the 151 unique genes, 98 were protein-coding genes, 8 were tRNA genes, and 45 were rRNA genes. The structural characteristics of the *R.mariesii*cp genome was similar to other angiosperms. Leucine was the most representative amino acid, while cysteine was the lowest representative. Totally, 30 codons showed obvious codon usage bias, and most were A/U-ending codons. Six highly variable regions were observed, such as *trnK-pafI* and *atpE-rpoB*, which could serve as potential markers for future barcoding and phylogenetic research of *R.mariesii* species. Coding regions were more conserved than non-coding regions. Expansion and contraction in the IR region might be the main length variation in *R.mariesii* and related Ericaeae species. Maximum-likelihood (ML) phylogenetic analysis revealed that *R.mariesii* was relatively closed to the *R.simsii* Planchon, 1853 and *R.pulchrum* Sweet,1831. This research will supply rich genetic resource for *R.mariesii* and related species of the Ericaeae.

## ﻿Introduction

*Rhododendronmariesii* Hemsley et Wilson, 1907, a typical member of the family Ericaeae, is mainly distributed in central China (Wang et al. 2018). Well known for leaf shape and bright-colored flowers, *R.mariesii* possesses valuable medicinal and horticultural properties (Wang et al. 2018). The deciduous species *R.mariesii* attracted great interest of *Rhododendron* breeders and geneticists. Furthermore, *R.mariesii* is very important in the woodland flora of the Dabie Mountains, and plays critical roles in ecological balance (Wang et al. 2018). Recently, *Rhododendron*-based ecological tourism, habitat fragmentation, and human activities have exerted significant effects towards natural growth of the wild *Rhododendron* population (Wang et al. 2019). Therefore, the research on population genetics and ecological conservation of wild *R.mariesii* is vital and necessary. However, limited genome information is available for *R.mariesii*, which has largely hindered corresponding genetic and molecular research.

In higher plants, the majority of plastomes are circular and quadripartite architecture consisting of two inverted repeat regions (IRa and IRb), a large single-copy region (LSC), and a small single-copy region (SSC) (Daniell et al. 2016; Hu et al. 2020; Abdullah et al. 2021). As maternally inherited organelle, the angiosperm plastome has a relatively conserved gene content and stable structure, which offers genetic markers sufficient for genome-wide evolutionary investigation at various taxonomic levels (Asaf et al. 2016; Zhang et al. 2017; Givnish et al. 2018). In plants, the size of cp genomes varied from 107 to 280 kb, containing approximately 130 genes related to photo synthesis and carbon fixation (Daniell et al. 2016; Rossini et al. 2021). The substitution rate of cp genome is lower than that of the nuclear genome, and 115–165 kb in cp genome is highly evolutionarily conserved (Smith 2015). However, specific genes exhibit accelerated evolution rates, such as *ycf1*, *matK*, and *rbcL*, which often serve as DNA barcoding (Dong et al. 2015; Wambugu et al. 2015; Zhang et al. 2017).

Next generation sequencing (NGS) has greatly increased the availability of genome data for non-species model, which facilitates the comparative cp genomics and phylogenetic studies at interspecific level (Santos and Almeida 2019; Pervez et al. 2022). In this research, the cpDNA of *R.mariesii* was assembled and annotated, SSR loci were characterized, comparative genomics and phylogenetic studies were also performed, hoping to benefit the studies of population evolution and conservation genetics of *R.mariesii* and related species.

## ﻿Material and methods

### ﻿Materials sampling and DNA extraction

Young and disease-free leaves of wild *R.mariesii* were sampled from the Dabie Mountains (central China, 29°16.13'N, 115°27.07'E, 1,005 m), dried in silica, and stored at -20 °C until further usage. In particular, sample collection was authorized by the Biodiversity Conservation of Huanggang Normal University. The specimens were identified by Hongjin Dong (Huanggang Normal University), who possesses a doctoral degree in botany. All materials were well conserved in the Huanggang Normal University Herbarium (Hubei province, China). Total genomic DNA was extracted and purified from fresh leaves according to Wang et al. (2019). Subsequently, the quality of total genome DNA was verified in 1% agarose gel stained by GelRed and quantified by spectophotometer (NanoDrop 1000, Thermofisher Scientific, USA).

### ﻿Genome sequencing, assembly, and annotation

Nextera DNA library preparation kit was used to construct the paired-end Illumina libraries. These libraries were sequenced on Illumina NovaSeq6000 Sequencing System (Illumina, Hayward, CA) in a paired-end run (500 cycles, 1 × 250 pb). After trimming adapter sequences and removing low-quality sequences, raw data was filtered by SOAPnuke software (Chen et al. 2018). Then, the high-quality reads were *de novo* assembled by GetOrganelle pipeline (Jin et al. 2020). BOWTIE2 were used to validate the assembled sequence error of *R.mariesii*cp genome through mapping raw sequencing reads to the assembled plastome (Hanussek et al. 2021). Online program Organelle Genome DRAW (OGDRAW) was used to draw the physical map of *R.mariesii*cp genome (Greiner et al. 2019). Furthermore, gene annotation and analysis were carried out with DOGMA and C_P_GAVAS softwares, respectively (Liu et al. 2012). The final annotations were also manually verified by Geneious (ver.8.0.2) (Yu et al. 2022). The cp genome data had been submitted to the National Center for Biotechnology Information (NCBI) database (https://www.ncbi.nlm.nih.gov/).

### ﻿Codon usage and nucleotide diversity analysis

Codon usage frequency was analyzed by CodonW software (https://sourceforge.net/projects/codonw/). Particularly, all protein coding genes were used for analysis. Relative synonymous codon usage (RSCU) analysis was carried out to measure codon usage bias (Rossini et al. 2021). The RSCU referred to the ratio of observed frequency of codons to frequency expected in regarding to the equal usage of synonymous codons for a certain amino acid (Rossini et al. 2021). In particular, RSCU value more than 1 means a preferred codon, otherwise the value less than and equal to 1 are considered as no codon usage bias (Morton 2022).

In total, eleven full chloroplast genomes of genus *Rhododendron* were downloaded from NCBI database: *R.molle* Siebold et Zuccarini, 1846; *R.griersonianum* Balfour filius et Forrest, 1919; *R.pulchrum* (Sweet) George Don, 1834; *R.henanense* Fang, 1983; *R.micranthum* Maximowicz, 1870; *R.delavayi* Franchet, 1886; *R.concinnum* Hemsley, 1890; *R.simsii* Planchon, 1876; *R.platypodum* Diels, 1990; *R.datiandingense* Feng, 1996; and *R.kawakamii* Hayata, 1911. Unique genes of these ten downloaded and the newly assemble *R.mariesii*cp genomes were extracted with PHYLOSUITE v1.2.2 and aligned by Windows version of MAFFT software, then nucleotide diversity (Pi) was calculated for each unique gene with DNASP ver 6.12.03 (Rossini et al. 2021).

### ﻿Simple sequence repeats (SSR) analysis

MISA software (MicroSAtellite identification tool v2.1, http://pgrc.ipk-gatersleben.de/misa) was used to identify SSR motifs. Minimum number of tandem repeat units were set as follows: five repeat units for tri-, tetra-, penta-, and hexanucleotide SSRs; six repeat units for di-nucleotide SSRs; 10 repeat units for mono-nucleotide SSRs. The maximal number of bases interrupting two SSRs in a compound microsatellite was 100 bp.

### ﻿Phylogenetic analysis

Through searing NCBI database, 21 cp genomes of Ericaceae species were found and downloaded: 12 species of *Rhododendron*; two species of *Vaccinium* Linnaeus, 1753; *Arbutusunedo* Sims, 1822; *Hemitomescongestum* Asa Gray, 1858; *Allotropavirgata* Torrey et Gray, 1868, *Monotropahypopitys* Linnaeus, 1753; *Pityopuscalifornicus* (Eastwood) H.F.Copeland, 1935; and 2 species of *Gaultheria* Kalm, 1753. Together with the newly assembled *R.mariesii*cp genome, these 22 cp genomes were used to construct phylogeny tree. These cp genomes were initially aligned with MAFFT for phylogenetic analysis (Yu et al. 2020). RAxML (version 8.2.8 for Windows) was used to run maximum likelihood (ML) analysis with a bootstrap value of 1000 (Alexandros 2014). FIGTREE v1.4 was used to visualize and adjust the ML trees (Yu et al. 2022). In particular, cp genome of *Pyrolarotundifolia* Benth. (1840) played the roles of an out-group.

### ﻿Comparative analysis of genome structure

The structural characteristics of cp genomes, containing newly assembled *R.mariesii* and 10 cp genomes of the genus *Rhododendron* (*R.delavayi*, *R.henanense*, *R.micranthum*, *R.concinnum*, *R.griersonianum*, *R.simsii*, *R.kawakamii*, *R.molle*, *R.platypodum*, and *R.datiandingense*) were compared and analyzed with mVISTA online tool (using Shuffle-LAGAN alignment program). In particular, the annotated cp genome of *R.mariesii* served as a reference against the other cp genome. Genome alignments, including rearrangements or inversions, was detected with MAUVE (Darling et al. 2004). For investigating whether expansion or contraction occurred in *R.mariesii*cp genome, the IR/LSC and IR/SSC junction regions were compared with IRscope software (Amiryousefi et al. 2018).

## ﻿Results

### ﻿General features of *R.mariesii* chloroplast genome

**Figure 1.**
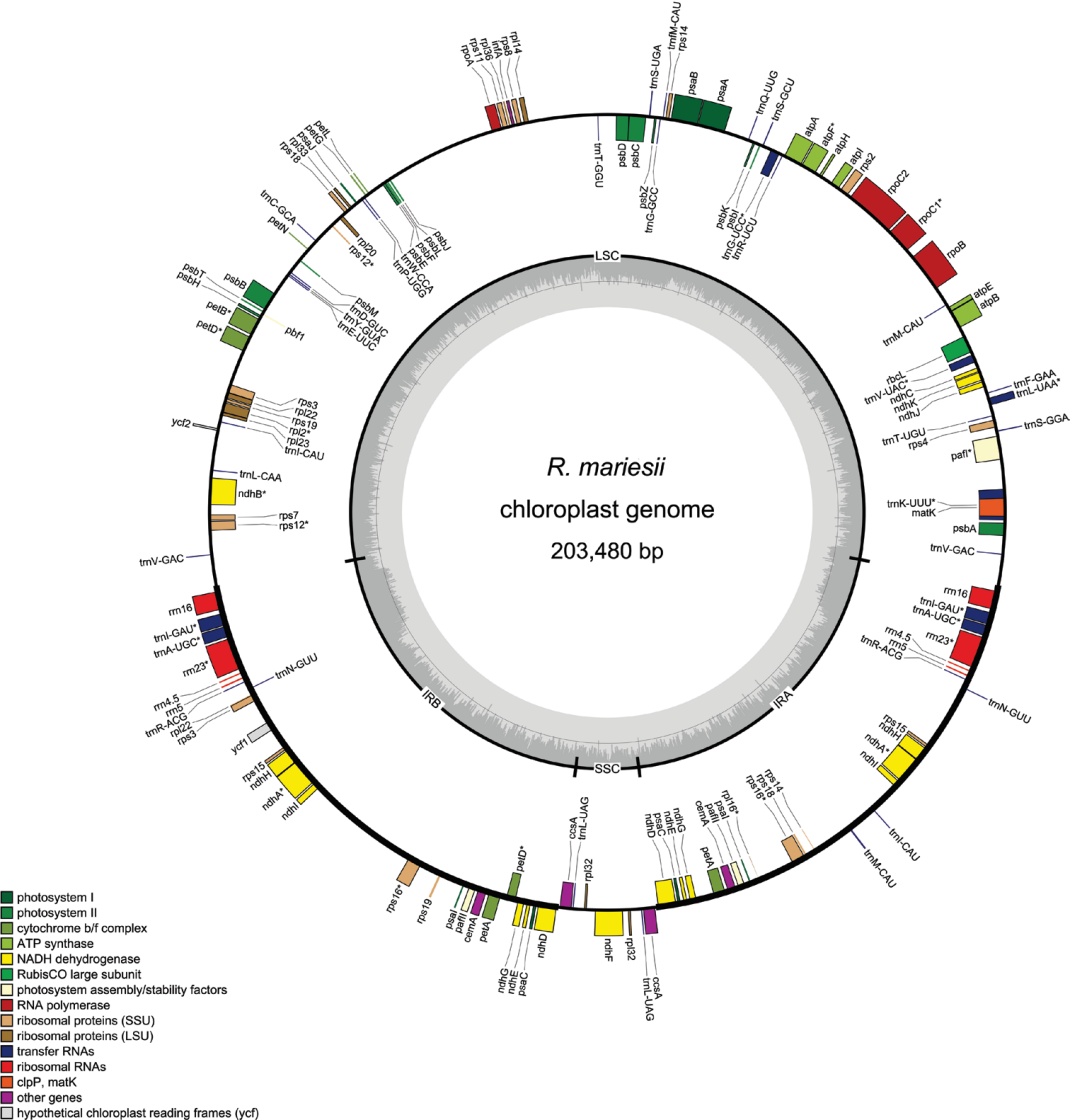
The chloroplast genome map of *R.mariesii*. Thick lines represented LSC, SSC, and IR regions, respectively. Genes shown inside circle were transcribed counterclockwise, and the outside outer circle were transcribed clockwise. Different gene groups were represented by different colors.

In total, 19,498,900 reads were obtained from NovaSeq paired-end run. After stringent quality assessment and filtering, 19,309,162 clean reads (2.891 Gb) with an average of 149 bp read length were obtained. The percentage of clean reads was 99.03%, and the clean bases were 2,891,089,781 bp. In particular, GC content was 39.52%. In addition, Q20 (a base with quality value greater than 20) and Q30 (a base with quality value greater than 20) values were 97.28% and 92.34%, respectively. The size of *R.mariesii*cp genome is 203,480 bp. Moreover, typical quadripartite structure was observed, as a large single-copy (LSC) region (113,715 bp) and a small single-copy (SSC) region (7,953 bp) were separated by a pair identical inverted repeat regions (IRs) (40,918 bp) (Fig. 1).

In total, 151 genes were successfully annotated, including 98 protein-coding genes, 45 tRNA genes, and 8 rRNA genes. The lengths of CDS, rRNA, tRNA, intergenic regions, and introns were 65,889 bp (32.38%), 8,998 bp (4.42%), 3,449 bp (1.7%), 45,409 bp (22.32%), and 80,033 bp (39.33%), respectively. The GC content of CDS, rRNA, tRNA, intergenic regions, and intron were 37.67%, 54.87%, 51.49%, 32.06%, and 33.75%, respectively. A set of 55 photosynthesis-related genes were found, containing six subunits of ATP synthase (*atpA*, *atpB*, *atpE*, *atpF*, *atpH*, and *atpI*), seven subunits of photosystem I, 17 subunits of photosystem II, 17 subunits of NADH-dehydrogenase, seven subunits of cytochrome b/f complex, and one subunit of rubisco (*rbcL*) (Table 1). Considering the self replication, 11 genes were large subunits of ribosome, four genes were DNA-dependent RNA polymerase, one gene was translational initiation factor, eight genes were rRNA genes, 45 genes were tRNA genes, and 18 genes were small subunit of ribosome (Table 1). The other genes were related to acetyl-CoA carboxylase, c-type cytochrome synthesis gene, envelope membrane protein, and maturase (Table 1). In addition, there were three conserved open reading frames, including one *ycf3* and two *ycf4*. Totally, 16 genes contained introns, containing *trnK-UUU*, *ycf3*, *trnL-UAA*, *trnV-UAC*, *ropB*, *atpF*, *trnS-CGA*, *accD*, *rpl16*, *ndhB*, *trnE-UUC*, *trnA-UGC*, *ndhA*, *trnA-UGC*, and *trnE-UUC* (Table 2). Besides *ycf3* and *accD* genes (three exons and two introns), the other 14 genes all had two exons and one intron.

**Table 1.** Gene content of *R.mariesii* chloroplast genome. The duplicated genes were included into brackets.

Category of genes	Group of genes	Name of genes
Genes for photosynthesis	Subunits of ATP synthase	*atpA*, *atpB*, *atpE*, *atpF*, *atpH*, *atpI*
	Subunits of photosystem I	*psaA*, *psaB*, *psaC* (2×), *psaI* (2×), *psaJ*
	Subunits of photosystem II	*psbA*, *psbB*, *psbC*, *psbD*, *psbE*, *psbF*, *psbH*, *psbI*, *psbJ* (3×), *psbK*, *psbL*, *psbM*, *psbN*, *psbT*, *psbZ*
	Subunits of NADH-dehydrogenase	*ndhA* (2×), *ndhB*, *ndhC*, *ndhD* (2×), *ndhE* (2×), *ndhF*, *ndhG* (2×), *ndhH* (2×), *ndhI* (2×), *ndhJ*, *ndhK*
	Subunits of cytochrome b/f complex	*petA* (2×), *petB*, *petD*, *petG*, *petL*, *petN*
	Subunit of rubisco	*rbcL*
Self replication	Large subunit of ribosome	*rpl2*, *rpl14*, *rpl16*, *rpl20*, *rpl22* (3×), *rpl32* (2×), *rpl33*, *rpl36*
	DNA dependent RNA polymerase	*rpoA*, *rpoB*, *rpoC1*, *rpoC2*
	Translational initiation factor	*infA*
	Ribosomal RNA genes	*rrn5S* (2×), *rrn*16S (4×), *rrn*23S (2×)
	Transfer RNA genes	*trnK-UUU*, *trnH-GUC*, *trnS-GGA*, *trnT-UGU* , *trnT-GGU*, *trnL-UAA*, *trnL-CAA*, *trnL-UAG* (2×), *trnM-CAU* (12×), *trnF-GAA*, *trnV-UAC*, *trnV-GAC* (2×), *trnR-UCU*, *trnR-ACG* (2×), *trnS-CGA*, *trnS-GCU*, *trnS-UGA*, *trnQ-UUG*, *trnW-CCA*, *trnP-UGG*, *trnC-GCA*, *trnD-GUC*, *trnY-GUA*, *trnE-UUC* (2×), *trnA-UGC* (2×), *trnN-GUU*, *trnA-UGC*, *trnE-UUC* (2×)
	Small subunit of ribosome	*rps2*, *rps3* (3×), *rps4*, *rps7*, *rps8*, *rps11*, *rps14*, *rps15* (3×), *rps16*, *rps18* (2×), *rps19* (3×)
Other genes	Subunit of acetyl-CoA-carboxylase	*accD*
	c-type cytochrom synthesis gene	*ccsA* (2×)
	Envelop membrane protein	*cemA* (2×)
	Maturase	*matK*
Unkown function	Conserved open reading frames	*ycf3*, *ycf4* (2×)

**Table 2.** The characteristics list of genes possessing introns.

Gene	Strand	Start	End	ExonI	IntronI	ExonII	IntronII	ExonIII
*trnK-UUU*	-	1,834	4,404	37	2499	35		
*ycf3*	-	6,794	8,753	124	711	232	742	151
*trnL-UAA*	+	11,313	11,909	35	512	50		
*trnV-UAC*	-	15,031	15,692	39	588	35		
*rpoB*	+	21,836	25,719	3,169	677	38		
*atpF*	+	35,554	36,820	161	700	406		
*trnS-CGA*	-	38,853	39,609	31	666	60		
*accD*	+	55,356	56,894	571	159	150	54	605
*rpl16*	-	59,253	167,784	9	108,121	402		
*ndhB*	-	101,387	103,550	721	685	758		
*trnE-UUC*	+	112,521	113,535	32	943	40		
*trnA-UGC*	+	113,600	114,490	37	818	36		
*ndhA*	+	126,079	128,272	563	1090	541		
*ndhA*	-	181,938	184,131	563	1090	541		
*trnA-UGC*	-	195,720	196,610	37	818	36		
*trnE-UUC*	-	196,675	197,689	32	943	40		

### ﻿Codon usage analysis and nucleotide diversity analysis

**Figure 2.**
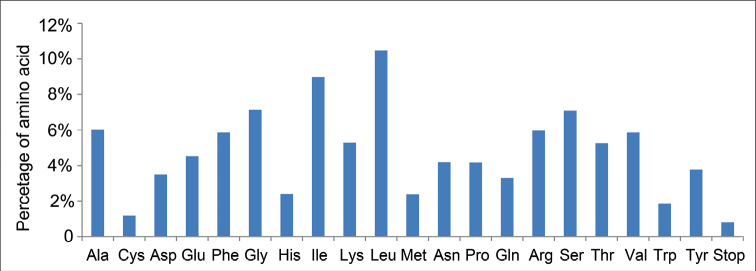
Occurrence percentage of amino acids in *R.mariesii* chloroplast genome.

In the *R.mariesii* chloroplast genome, the protein-coding regions presented 40,013 codons (Table 3). Particularly, leucine (Leu) was the main amino acid (10.477%), followed by isoleucine (Ile, 8.972%) and glycine (Gly, 7.148%) (Fig. 2). In particular, cysteine (Cys) and tryptophan (Trp) were the lowest representative amino acids, accounting for 1.180% and 1.869%, respectively. According to RSCU values, a total of 30 codons showed obvious codon usage bias, as RSCU value were more than 1 (Table 3). Except Leu codon (UUG), all the other 29 codons were A/U-ending. For the 34 codons with RSCU values less than 1, 31 were C/G-ending, while 3 were A/U-ending.

**Table 3.** The relative synonymous codon usage in *R.mariesii*cp genome.

Amino acid	Codon	No	RSCU	The codon frequency per amino acid(%)	Amino acid	Codon	No	RSCU	The codon frequency per amino acid(%)
Ala	GCA	703	1.17	29.16	Pro	CCA	509	1.22	30.55
	GCC	345	0.57	14.31		CCC	294	0.71	17.65
	GCG	275	0.46	11.41		CCG	202	0.48	12.12
	GCU	1088	1.81	45.13		CCU	661	1.59	39.67
Cys	UGC	118	0.5	24.99	Gln	CAA	1053	1.59	79.65
	UGU	354	1.5	74.97		CAG	269	0.41	20.35
Asp	GAC	273	0.39	19.57	Arg	AGA	653	1.64	27.37
	GAU	1122	1.61	80.44		AGG	179	0.45	7.5
Glu	GAA	1395	1.54	77.2		CGA	626	1.57	26.24
	GAG	412	0.46	22.8		CGC	147	0.37	6.16
Phe	UUC	753	0.64	32.19		CGG	158	0.4	6.62
	UUU	1586	1.36	67.8		CGU	623	1.57	26.11
Gly	GGA	1079	1.51	37.73	Ser	AGC	188	0.4	6.61
	GGC	327	0.46	11.43		AGU	580	1.22	20.41
	GGG	465	0.65	16.26		UCA	507	1.07	17.84
	GGU	989	1.38	34.58		UCC	415	0.88	14.6
His	CAC	223	0.47	23.28		UCG	240	0.51	8.44
	CAU	735	1.53	76.73		UCU	912	1.93	32.09
Ile	AUA	1125	0.94	31.34	Thr	ACA	640	1.22	30.39
	AUC	674	0.56	18.78		ACC	411	0.78	19.52
	AUU	1791	1.5	49.89		ACG	213	0.4	10.11
Lys	AAA	1631	1.54	77.11		ACU	842	1.6	39.98
	AAG	484	0.46	22.88	Val	GUA	845	1.44	36.11
Leu	CUA	518	0.74	12.36		GUC	302	0.52	12.91
	CUC	251	0.36	5.99		GUG	319	0.55	13.63
	CUG	248	0.35	5.92		GUU	874	1.49	37.35
	CUU	889	1.27	21.21	Trp	UGG	748	1	100.02
	UUA	1475	2.11	35.18	Tyr	UAC	306	0.41	20.32
	UUG	811	1.16	19.35		UAU	1200	1.59	79.68
Met	AUG	954	1	100.01	Stop*	UAA	133	1.22	40.69
Asn	AAC	382	0.46	22.78		UAG	88	0.81	26.92
	AAU	1295	1.54	77.22		UGA	106	0.97	32.42

**Figure 3.**
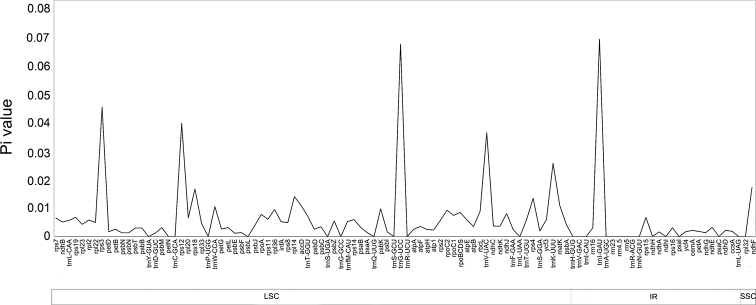
The nucleotide diversity (Pi) of 11 *Rhododendron* species chloroplast genomes. X-axis presented the position of aligned chloroplast genomes, and Y-axis referred to Pi value. Below the X-axis, large single-copy (LSC), small single-copy (SSC), as well as inverted repeat (IR) regions were displayed with arrow bars.

Nucleotide diversity analysis showed that sequence level of divergence existed between different *Rhododendron*cp genomes. Pi values for each gene region varied from 0 to 0.06896. High level of genetic variation mainly existed in SSC region (Pi = 0.01723), followed by LSC (Pi = 0.00697) and IR (Pi = 0.001224) regions (Fig. 3). In total, six gene regions showed high levels of nucleotide diversity (Pi > 0.02), containing *trnI-GAU* (Pi = 0.06896), *trnG-UCC* (Pi = 0.06721), *rps3* (Pi = 0.04509), *rps12* (Pi = 0.03947), *trnV-UAC* (Pi = 0.03622), and *trnK-UUU* (Pi = 0.02554).

### ﻿SSR analysis of *R.mariesii* plastome

A set of 70 SSRs were identified from *R.mariesii*cp genome, and 5 SSRs were present in compound formation. Particularly, 65 SSRs (92.86%) were mononucleotide motifs, 2 were dinucleotide motifs (2.86%), 2 were trinucleotide motifs (2.86%), and 1 were hexanucleotide repeats (1.43%) (Table 4). Dominant mononucleotide repeats were A/T (91.429%), while C/G repeats accounted for 1.429%. In related to dinucleotide motifs, only AT/AT type was found (2.857%). For trinucleotide motifs, only one (AAG/CTT)5 and one (AAT/ATT) 11 motif were found. For hexanucleotide repeats, one (AAGGGT/ACCCTT)5 was found, accounting for 1.429%.

**Table 4.** The frequency of each type of microsatellite in *R.mariesii*cp genome.

Repeats	5	6	7	8	9	10	11	12	13	14	15	total	Percentage
A/T	-	-	-	-	-	32	14	9	7	2		64	91.429%
C/G	-	-	-	-	-						1	1	1.429%
AT/AT	-		2									2	2.857%
AAG/CTT	1											1	1.429%
AAT/ATT							1					1	1.429%
AAGGGT/ACCCTT	1											1	1.429%

Mononucleotide A/T repeats with repeat numbers of 10–14 were the most abundant. Meanwhile, (C/G)n microsatellites were all repeated 15 times. In relation to dinucleotide repeats, the identified SSRs all have 7 repeat motifs. Regarding to trinucleotide motifs, AAG/CTT and AAT/ATT microsatellites repeated 5 and 11 times, respectively. The hexanucleotide motif AAGGGT/ACCCTT repeated 5 times. Totally, 34 SSRs were present in the intergenic spacer region, accounting for 41.43%. Moreover, 28 SSRs were present in *rpl16* gene. All the remaining 13 microsatellites were found in *ccsA*, *cemA*, *ndhA*, *rpoA*, *rpoC2*, *rps7*, *rps8*, and *trnL-UAA* genes.

### ﻿Phylogenetic analysis

**Figure 4.**
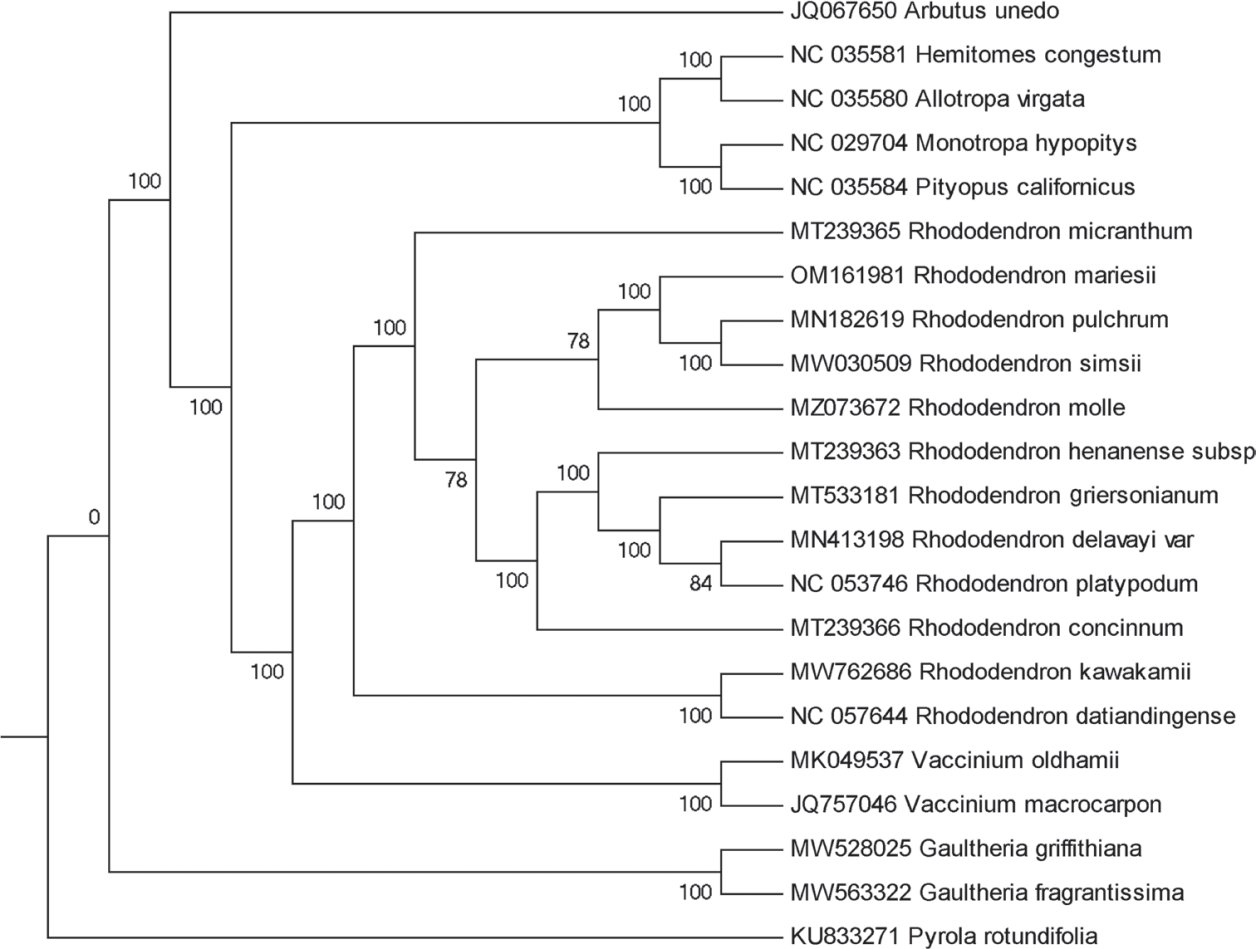
The maximum-likelihood phylogenetic tree for *R.mariesii*. Numbers on each node referred to bootstrap support value.

For clarifying the phylogenetic location of *R.mariesii* among the Ericaeae, complete plastomes of *R.mariesii* and other 21 species in the Ericaeae with fully sequenced chloroplast genomes were used in reconstructing phylogenetic relationships. The phylogenetic tree revealed that *R.mariesii* had a close genetic relationship with *R.simsii* and *R.pulchrum* (Fig. 4). In particular, all these 22 taxa belonging to Ericaeae were grouped into one clade and clustered into two subclades. Topological structure was almost consistent with the previously published phylogeny (Liu et al. 2021). *A.unedo* (NCBI: JQ067650), *H.congestum* (NCBI: NC_035581), *A.virgate* (NCBI: NC_035580), *M.hypopitys* (NCBI: NC_029704), *P.californicus* (NCBI: NC_035584), *Vacciniumoldhamii* Miquel, 1866 (NCBI: MK049537), *Vacciniummacrocarpon* Aiton, 1789 (NCBI: JQ757046), *Gaultheriagriffithiana* Wight, 1847 (NCBI: MW528025), *Gaultheriafragrantissima* Wallich, 1820 (NCBI: MW563322), and *P.rotundifolia* (NCBI: KU833271), were relatively distant related with *R.mariesii*.

### ﻿Comparative plastome sequence divergence and hotspot regions

**Figure 5.**
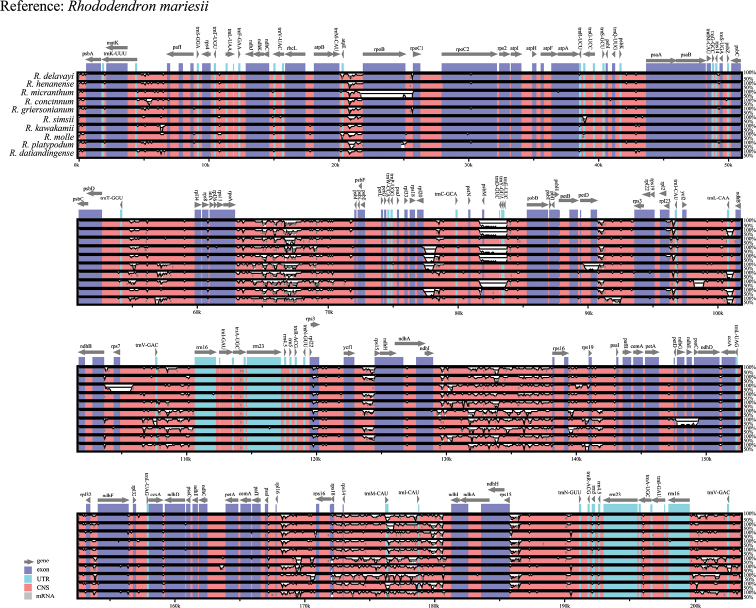
Comparison of cp genomes with *R.mariesii* annotation serving as the reference. Vertical scale indicated the percentage of identity (50–100%), and horizontal axis was coordinates within cp genome. The genome regions were color-coded as exons, introns, and conserved non-coding sequences, respectively.

Structural characteristics of 11 *Rhododendron*cp genomes were investigated with mVISTA software, containing the newly assembled *R.mariesii*cp genome and 10 download cp genome of the *Rhododendron* genus. In particular, the annotated *R.mariesii*cp genome served as a reference. Relatively high similarity was detected among these 11 *Rhododendron* species. Coding regions were more conserved than non-coding regions (CNS in Fig. 5). The LSC and SSC regions were relatively more stable than IR regions. Among these coding regions, *rpoB*, *rpoC2*, *rps8*, *petD*, *rpl23*, *rpl22*, and *ndhF* were relatively divergent because of intron regions. In *R.mariesii* plastid genome, highly variable regions mostly existed in the intergenic spacer, such as *trnK-pafI*, *atpE-rpoB*, *trnT-rpl14*, *rpoA-psbJ*, *rpl20*-*trnE*, *ndhI*-*rps19*, and *rpl16*-*ndhI*. Compared with intergenic spacer, protein coding regions were highly conserved, such as *rps4*, *ndhJ*, *ndhK*, *rpcL*, rps2, atpI, *psaA*, *psaB*, *psbB*, *cemA*, and *petA*. No rearrangements and inversions occurred in these 11 cp genomes of *Rhododendron* species.

**Figure 6.**
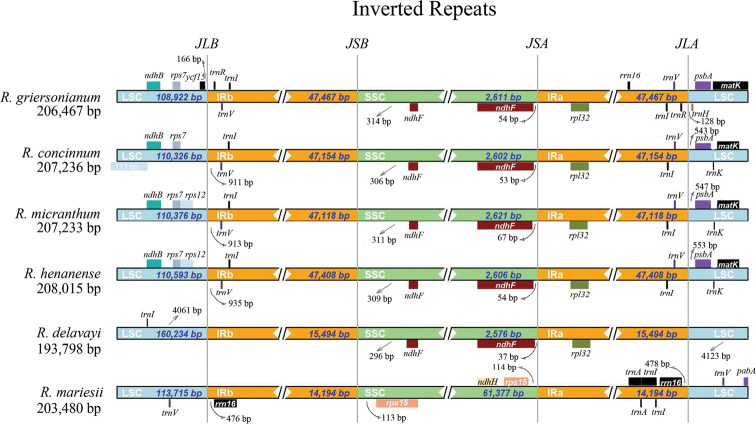
The comparison of LSC, SSC, and IR regional boundaries of cp genome between *R.mariesii* and related taxa. JLB, JSB, JSA, and JLA respected “junction line between LSC and IRb”, “junction line between IRb and SSC”, “junction line between SSC and IRa” , as well as “junction line between IRa and LSC”, respectively.

Particularly, lengths of the IR regions of 6 cp genomes ranged from 14,194 bp (*R.mariesii*cp genome) to 47,467 (*R.griersonianum*cp genome) (Fig. 6). Expansion and contraction existed in these cp genomes. In *R.griersonianum*cp genome, JLB line (line between LSC and IRb) was located between genes *ycf15* and *trnR*, while *ycf15* was located in the LSC region with 166 bp extending to IRb region. In *R.mariesii*cp genome, JLB line was located between *trnV* and *rrn16* (476 bp extending to LSC region). In *R.micranthum* and *R.henanense*cp genomes, the JLB lines were located between *rps12* and *trnV*, while *trnV* was located in IRb region with 913 bp and 935 bp extending to LSC region, respectively. However, JLB line was located between *rps7* and *trnI*, and *trnI* was located in IRb region with 911 bp extending to LSC region in *R.concinnum*. Except for *R.mariesii*, *ndhF* was located in SSC region with 296 bp–314 bp extending to IRb region in the five other cp genomes (Fig. 6). Meanwhile, *rps15* was located in SSC region of *R.mariesii*cp genome. The JSA line (line between SSC and IRa) was located between *ndhF* and *rpl32*, and *ndhF* was distributed in SSC region with 54 bp, 53 bp, 67 bp, 54 bp, and 37 bp to IRa region in *R.griersonianum*, *R.concinnum*, *R.micranthum*, *R.henanense*, and *R.delavayi*cp genomes, respectively. Besides *ndhH*, *rps15* was also located in SSC region with 114 bp extending to IRa region in *R.mariesii*cp genome. Furthermore, JLA line (line between IRa and LSC) was located between *trnV* and *psbA* in *R.concinnum*, *R.micranthum*, and *R.henanense*. However, JLA line was located between *trnR* and *trnH* in *R.griersonianum*cp genome. In *R.mariesii*cp genome, *rrn16* and *trnV* were located besides the JLA line.

## ﻿Discussion

The chloroplast genome is the main organelle for plant transforming light energy into chemical energy (Zhang et al. 2018). Plastome genome is useful for comparative genomic research and phylogenomic analyses due to polymorphic regions generated through genomic expansion, inversion, contraction, and gene rearrangement (Sanitá Lima et al. 2016; Kahraman et al. 2019; Li et al. 2019; Li et al. 2020; Liu et al. 2020; Wang et al. 2020). The single circular cp genome structure of *R.mariesii* was the same as other species belonging to the Ericaeae with a typical quadripartite structure and similar GC content unevenly distributed across the cp genome (Liu et al. 2021; Xu et al. 2022). The GC content of *R.mariesii*cp genome (39.52%) was slightly larger than that of *Myracrodruonurundeuva* Allemão, 1862 (37.8%) (Rossini et al. 2021). Relative to both LSC (35.85%) and SSC (36.49%) regions, the GC content in IR region (30.48%) is lower. However, the GC content in IR region was larger than LSC and SSC regions in cp genome *Xanthiumspinosum* Linnaeus, 1753 (Raman et al. 2020). The length of total genome size and each region were similar to other plant cp genomes, such as *R.molle*, *Rubus* species (Rosaceae), and rubber dandelion (*Taraxacumkok-saghyz* Rodin) (Zhang et al. 2017; Xu et al. 2022; Yu et al. 2022).

The size of *R.mariesii*cp genome (203,480 bp) was larger than that of *R.pulchrum* (146,941 bp), *R.simsii* (152,214 bp), *R.molle* (197,877 bp), *R.delavayi* (193,798 bp), and *R.platypodum* (201,047 bp), but smaller than that of *R.kawakamii* (230,777 bp), *R.micranthum* (207,233 bp), *R.henanense* (208,015 bp), *R.griersonianum* (206,467 bp), *R.concinnum* (207,236 bp), and *R.datiandingense* (207,311 bp). Totally, 151 genes existed in *R.mariesii*cp genome, which were more than that of *R.molle* (149 genes) and *R.pulchrum* (73 genes) (Shen et al. 2020; Xu et al. 2022). In particular, protein-coding genes accounted for 64.901% in *R.mariesii*cp genome, which were lower than that of *R.molle*cp genome (65.101%) but higher than that of *R.pulchrum*cp genome (57.534%) (Shen et al. 2020; Xu et al. 2022). Moreover, 45, 44, and 29 tRNA genes were found in cp genomes of *R.mariesii*, *R.molle*, and *R.pulchrum*, respectively. In both *R.mariesii* and *R.molle*cp genomes, eight rRNA genes were annotated, but only two were found in *R.pulchrum*cp genome (Shen et al. 2020; Xu et al. 2022).

Besides genes involved in photosynthesis transforming light energy into chemical energy, other genes also existed in *R.mariesii*cp genome. For example, *accD* gene, encoding plastid beta carboxyl transferase subunit of acetyl-CoA carboxylase (ACCase) important for plant growth (leaf growth, leaf longevity, fatty acid biosynthesis, and embryo development), has been reported to be involved in the adaptation to specific ecological niches during radiation of dicotyledonous plants (Hu et al. 2015). In *R.mariesii*cp genome, one copy of *accD* gene was also found. Codons coding Leu (10.477%), Ile (8.972%), and Gly (7.148%) were dominant, while Cys (1.180%) and Trp (1.869%) were the least, which were the same as that of *M.urundeuva*cp genome (Rossini et al. 2021). Codon bias, an efficient mechanism of translation influenced by natural selection and mutation pressure, takes place if synonymous codons are used at different frequencies (Zhang et al. 2022). A total of 30 codons showed codon usage bias, and most were A/U-ending codons, which were the same as that observed in *M.urundeuva* and *Solanum* (Zhang et al. 2018a; Rossini et al. 2021). In total, six gene regions showed high levels of nucleotide diversity (Pi > 0.02), containing *trnI-GAU*, *trnG-UCC*, *rps3*, *rps12*, *trnV-UAC*, and *trnK-UUU*, serving as the first candidate for developing molecular markers to identify *Rhododendron* species.

A total of 70 SSRs were identified from *R.mariesii*cp genome, more than that of *M.urundeuva* (36 SSRs), *Spondiasbahiensis* P. Carvalho, 2015 (53 SSRs) and *Mangiferaindica* Wallich, 1847 (57 SSRs), but fewer than that of *Syringapinnatifolias* Hemsley, 1906 (253 SSRs) (Jo et al. 2017; Santos and Almeida 2019). Variation in the number and type of microsatellites might play important roles in plastome organization. The main motifs were A/T repeats (91.429%), which was the same with that of *M.urundeuva*, *S.bahiensis*, and *M.indica* (Jo et al. 2017; Santos and Almeida 2019; Rossini et al. 2021). However, no correlation was found between large repeat regions and rearrangement endpoints, which was similar with Liu et al. (2013). Very limited tandem (G/C)n-containing microsatellites were observed, which might be due to the low content of G and C bases in chloroplast genome. Molecular markers developed for the intergenic regions could be used for phylogenetic, phylogeographic, and barcoding studies of *Rhododendron* species.

Non-coding regions often mutate relatively faster than coding regions (Yu et al. 2022). In *R.mariesii*cp genome, coding regions were more conserved than the non-coding regions. Relatively high similarity was detected among these *Rhododendron*cp genomes, but expansion and contraction also existed in IR regions, which might be the dominant reason for variation in cp genome size. Obvious differences were found in cpIR boundary regions, containing gene contents and locations. However, IR regions were least divergent, which were mainly due to the presence of four highly conserved rRNA sequences in *X.spinosum* (Raman et al. 2020). Furthermore, LSC and SSC regions were relatively more stable than IR regions in *R.mariesii*cp genome. These genetic variations may significantly facilitate *R.mariesii* adapting to the changes of survival conditions. According to neutral theory, nucleotide substitution in non-coding regions (intergenic spacer, intron region, and pseudogenes) are neutral or near-neutral, which could not be affected by natural selection (Akashi et al. 2012). Therefore, evolutionary history of *R.mariesii* could be well calculated from the rate of molecular evolution in non-coding region.

This research aimed to expand the molecular genetic resources available for *R.mariesii* through high-throughput sequencing and cp genome assembly. The *R.mariesii*cp genome sequence could be used in distinguishing and resolving phylogenetic relationships within Ericaeae lineage. Moreover, this research will be vital for further genetic analysis on *R.mariesii* and other species in the Ericaeae family.

## ﻿Conflicts of interests

The authors declare that they have no competing interests.

## ﻿Data availability statement

The cp genome of *R.mariesii* was submitted to GenBank database under the accession number of OM161981.

## Supplementary materials

Supplementary material 1 Taxonomic and accession information on cp genomes downloaded from NCBI database Data type wps https://binary.pensoft.net/file/893823This dataset is made available under the Open Database License (http://opendatacommons.org/licenses/odbl/1.0/). The Open Database License (ODbL) is a license agreement intended to allow users to freely share, modify, and use this Dataset while maintaining this same freedom for others, provided that the original source and author(s) are credited.Zhiliang Li, Zhiwei Huang, Xuchun Wan, Jiaojun Yu, Hongjin Dong, Jialiang Zhang, Chunyu Zhang, Shuzhen Wang
